# Unmet clinical needs in chronic migraine: Rationale for study and design of COMPEL, an open-label, multicenter study of the long-term efficacy, safety, and tolerability of onabotulinumtoxinA for headache prophylaxis in adults with chronic migraine

**DOI:** 10.1186/s12883-015-0353-x

**Published:** 2015-07-03

**Authors:** Andrew M. Blumenfeld, Sheena K. Aurora, Karen Laranjo, Spyros Papapetropoulos

**Affiliations:** The Neurology Center, 320 Santa Fe Drive, Suite 150, Encinitas, CA 92024 USA; Stanford University, 300 Pasteur Drive, Room A343, MC 5235, Stanford, CA 94305 USA; Formerly an employee of Allergan, Inc., Irvine, CA USA; University of Miami, Miller School of Medicine, Miami, FL USA

**Keywords:** onabotulinumtoxinA, chronic migraine, headache prophylaxis, rationale and design, COMPEL

## Abstract

**Background:**

Chronic migraine is a neurological condition with a large individual and socioeconomic burden of disease. The recently completed Phase III REsearch Evaluating Migraine Prophylaxis Therapy (PREEMPT) clinical development program established the efficacy and safety of onabotulinumtoxinA as a prophylactic treatment for chronic migraine patients. However, clinical questions remain. A long-term evaluation study of onabotulinumtoxinA aims to address some of the remaining questions in the treatment of chronic migraine. The clinical rationale, study design, and treatment plan of this ongoing study are reviewed in this paper.

**Methods/Design:**

The *C*hronic migraine *O*nabotulinu*M*toxinA *P*rolonged *E*fficacy open *L*abel (COMPEL) study will enroll approximately 500 adult patients with chronic migraine at international sites. Patients will be evaluated over 108 weeks, following a 4-week baseline period. Qualified subjects will receive 155 U of onabotulinumtoxinA every 12 weeks for 9 open-label cycles. The primary endpoint will be mean change from baseline in frequency of headache days at 108 weeks. Other endpoints will include additional assessments of the efficacy and safety of onabotulinumtoxinA and the effect of onabotulinumtoxinA on quality-of-life measures, disability, and health economic outcomes. The impact of onabotulinumtoxinA on common comorbidities (eg, sleep, anxiety, and fatigue) will also be assessed.

**Discussion:**

Recruitment and enrollment are ongoing. Post-approval, open-label studies are often designed to more closely resemble clinical practice and provide an opportunity to continue the evaluation of the efficacy and safety of approved treatments. By creating a large database and analyzing a variety of outcome measures over an extended time frame, the COMPEL study will seek to contribute substantially to the existing knowledge of the chronic migraine population and the long-term management of this debilitating disorder.

**Clinical Trial Registration Number:**

NCT01516892

## Background

Chronic migraine (CM) is a severe neurological disorder with a global prevalence of approximately 2 % of adults [1]. According to the International Headache Society, CM is defined as headache on 15 or more days per month for more than 3 months (at least 8 days should meet criteria for migraine without aura or respond to migraine-specific treatment) [1, 2]. Chronic migraine is less prevalent than episodic migraine (EM; headache on <15 days per month), but it has far-reaching individual and societal impact [1, 2].

Compared with EM patients, patients with CM are more likely to have severe disability and an inability to work, function within society, and manage household chores. Patients with CM report a lower health-related quality of life (HRQoL), greater lost productive time, and greater healthcare resource utilization than EM patients [3–6]. Additionally, psychiatric comorbidities such as depression and anxiety are more prevalent among chronic migraineurs than episodic migraineurs [7]. Other frequent comorbidities include fatigue and sleep disorders [8]. Given the severe burden of illness suffered by CM patients, as well as the comorbidities associated with the condition, it is important to design studies that aim to improve understanding of treatment effects for this debilitating disorder.

Currently, onabotulinumtoxinA is the only therapy specifically approved in the US for prophylaxis of headache in CM patients, and the treatment paradigm and dosing are specific to this product.

The currently understood mechanism of action of onabotulinumtoxinA is via interruption of the inflammatory cascade of events that results in peripheral nociceptor sensitization and, subsequently, central sensitization [9, 10]. With neuronal stimulation, the peripheral sensory nerve ending releases vesicles storing substance P, calcitonin gene-related peptide (CGRP), and other neuropeptides. The fusion of the vesicles with the nerve membrane requires the formation of the soluble N-ethylmaleimide-sensitive factor attachment protein receptor (SNARE) complex, which then facilitates the release of their cargo by the process of exocytosis [9, 11]. As demonstrated in preclinical studies [12], via receptor-mediated endocytosis, onabotulinumtoxinA enters an intracellular membranous endosome, translocates across the endosome into the cytoplasm and cleaves its SNARE protein target, synaptosomal-associated protein 25 (SNAP-25), which blocks the peripheral release of inflammatory neurotransmitters, which in turn reduces the sensitization of pain nerve fibers [9, 10].

The efficacy and safety of onabotulinumtoxinA in CM was evaluated over a 56-week period (24-week, double-blind period, followed by 32 weeks of open-label use) in the Phase III REsearch Evaluating Migraine Prophylaxis Therapy (PREEMPT) clinical program, the largest study in CM to date [13]. Compared to placebo, onabotulinumtoxinA was more effective in reducing mean frequency of headache days from baseline at every visit from Week 4 to the primary endpoint of Week 24 (−8.4 onabotulinumtoxinA vs. -6.6 placebo; p < 0.001) [13]. Statistically significant improvements from baseline in secondary efficacy variables (eg, frequency of migraine days and moderate or severe headache days, and cumulative hours of headache) were established in patients treated with onabotulinumtoxinA compared to placebo. Patients treated with onabotulinumtoxinA also had a significant reduction in severe (>60) Headache Impact Test-6™ (HIT-6) scores (p < 0.01) and experienced an improvement in HRQoL at all time points, as measured by the Migraine-Specific Quality-of-Life Questionnaire (MSQ) (p < 0.001) [13]. Most adverse events reported in the PREEMPT clinical program were mild to moderate, and all resolved without permanent sequelae [13].

The goal of the *C*hronic migraine *O*nabotulinu*M*toxinA *P*rolonged *E*fficacy open *L*abel (COMPEL) study is to test the following clinical hypotheses: (1) onabotulinumtoxinA is an efficacious form of headache prophylaxis in adults with CM, as measured by a reduction from baseline in the number of headache days over a 28-day period after 108 weeks (9 cycles) of treatment; and (2) onabotulinumtoxinA has an acceptable safety profile over 108 weeks (9 cycles) of treatment. Efficacy and safety measures will be assessed at Week 60 and Week 108. Table 1 summarizes the main objectives of COMPEL. Other exploratory hypotheses will be tested in the COMPEL trial, including whether: (1) onabotulinumtoxinA treatment is associated with improvement in depression (Patient Health Questionnaire [PHQ-9]), sleep (Pittsburgh Sleep Quality Index [PSQI]), anxiety (Generalized Anxiety Disorder Assessment [GAD-7]), and fatigue (Fatigue Severity Scale [FSS]); (2) the benefit of onabotulinumtoxinA treatment is observed in the presence of concomitant prophylactics; (3) onabotulinumtoxinA treatment may be associated with a significant decrease in disability and improvement in HRQoL; and (4) onabotulinumtoxinA treatment may be associated with increased productivity and reduced disability and utilization of healthcare resources by patients.

**Table 1:** Study objectives for COMPEL

Primary Objective
To assess mean change from baseline in the frequency of headache days per 28-day period at 108 weeks (following 9 treatments) using a patient diary completed via IVRS.
Secondary Objectives
To assess mean change from baseline in the frequency of headache days for the 28-day period ending at 60 weeks (following 5 treatments).
To assess the efficacy of onabotulinumtoxinA treatment for CM in adult patients as measured by the mean change from baseline in total HIT-6 score over a 4-week period at 108 weeks (following 9 treatments) and at 60 weeks (following 5 treatments).
To evaluate the long-term safety and tolerability (9 treatment cycles) of onabotulinumtoxinA for CM in adult patients.

CM = chronic migraine; HIT-6 = Headache Impact Test-6; IVRS = interactive voice response system

## Methods/design

### Study design

**Fig. 1:**
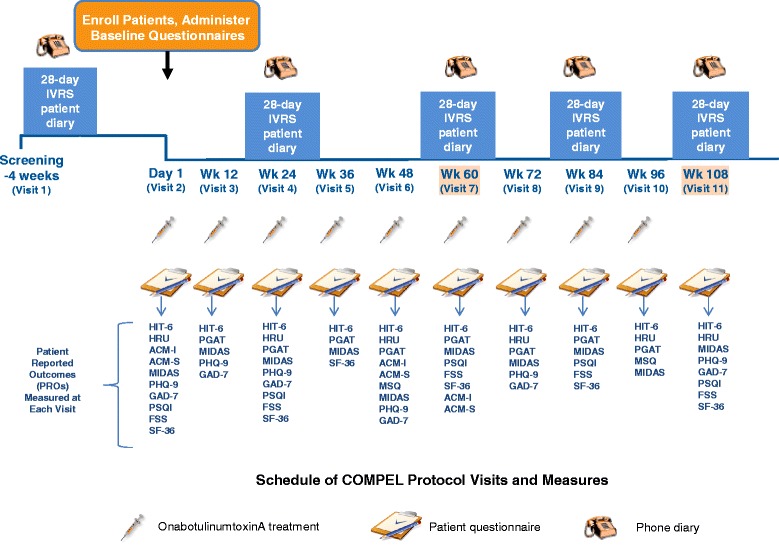
COMPEL study design. ACM-I = Assessment of Chronic Migraine Impacts; ACM-S = Assessment of Chronic Migraine Symptoms; FSS = Fatigue Severity Scale; GAD-7 = Generalized Anxiety Disorder Assessment; HIT-6 = Headache Impact Test-6; HRU = Healthcare Resource Utilization; IVRS = interactive voice response system; MIDAS = Migraine Disability Assessment Questionnaire; MSQ = Migraine-Specific Quality-of-Life Questionnaire v2.1; PHQ-9 = Patient Health Questionnaire-9; PSQI = Pittsburgh Sleep Quality Index; SF-36 = Short-Form 36 Health Survey; Wk = week

The COMPEL study is a single-arm, open-label, multicenter, post-authorization, prospective study of onabotulinumtoxinA use in CM for headache prophylaxis. The duration of the study is 112 weeks, including a 4-week baseline period and a 108-week, open-label treatment intervention phase (Fig. 1). There will be a total of 11 patient visits, including an initial baseline visit at −4 weeks (Visit 1), 9 interventional visits (on Day 1 and at Weeks 12, 24, 36, 48, 60, 72, 84, and 96), and a study exit visit at Week 108 (Visit 11). Baseline measures of the patient population will include demographics, medical history, physical exam, headache history, detailed headache treatment history, and headache features. Patient diaries will be collected via interactive voice response system (IVRS). Diary data will be entered daily during the 4-week baseline period after Visit 1 and then daily for 28 days, starting 4 weeks prior to Visits 4, 7, 9, and 11.

### Study eligibility criteria

Study entry screening will be limited to adult patients who are 18 years of age or older, have a diagnosis of CM, are in stable medical condition, are able to follow and comply with study instructions and required visits, and have a negative urine pregnancy test, if applicable. Patients participating in the study may take concurrent medications such as vitamins, herbal remedies, dietary supplements, and a single oral headache prophylaxis medication (ie, beta-blocker, calcium channel blocker, angiotensin-converting enzyme [ACE] inhibitor, aldosterone receptor blocker, antiepileptic, or tricyclic antidepressant), provided these medications are documented. Only one concomitant oral headache prophylaxis medication can be used at any time during the study. A second oral prophylaxis medication may not be added for any patient. All patients on oral headache prophylaxis medication may discontinue this treatment anytime during the study. Patients that discontinue oral prophylaxis may continue treatment only with onabotulinumtoxinA (no alternative oral headache prophylaxis can be added later) (Table 2).

**Table 2:** Criteria for use of oral headache prophylaxis* in COMPEL

Use of OHP	Subjects Taking Single OHP Prior to Visit 2 (Day 1)	Subjects Not Taking OHP Prior to Visit 2 (Day 1)
Continuation of OHP	Must be on stable dose and regimen of single OHP for ≥4 weeks prior to Visit 2 (Day 1)	—
Addition of OHP	—	OHP may be added after Visit 4 (Week 24)
Change of OHP Dose	Dose may be changed at or after Visit 4 (Week 24)	—
Discontinuation of OHP	May occur at any time during study, but cannot be replaced by same or other OHP	

*Only a single concomitant OHP allowed during the study, and may include tricyclic antidepressant, antiepileptic, beta-blocker, calcium channel blocker, angiotensin enzyme (ACE) inhibitor, or angiotensin receptor blocker (ARB)

OHP = oral headache prophylaxis medication

For patients who are taking oral headache prophylaxis medication, patient must be on a stable dose and regimen of a single oral prophylaxis treatment for at least 4 weeks prior to Visit 2 (Day 1). For patients on stable dose and regimen of a single oral headache prophylaxis medication for at least 4 weeks prior to Visit 2, the dose may be changed at or after Visit 4 (Week 24). Those patients who are not taking oral headache prophylaxis at study entry must have discontinued their oral prophylaxis more than 4 weeks prior to Visit 1 to be eligible for inclusion in the study. For patients not on oral headache prophylaxis at Visit 2, a single oral prophylaxis medication such as a beta-blocker, calcium channel blocker, ACE-inhibitor, aldosterone receptor blocker, antiepileptic or tricyclic antidepressant may be added to the patient’s regimen as a component of the headache prevention at or after Visit 4 (Week 24). If a patient is receiving a medication listed as a headache prophylactic that is specifically prescribed for a non‐headache condition, he or she is eligible to enter the study. They are also eligible if receiving another agent on the headache prophylactic list that has been prescribed for headache as long as the prophylactic medication regimen is stable for at least 4 weeks prior to Visit 1.

Patients may take abortive headache medications and may add, change, or decrease these medications as needed if recorded in the patient diary via IVRS. Medications that are both acute and prophylactic treatments will be classified on the basis of the indication for use: if they are specifically prescribed for daily use for headache prevention, they are considered to be prophylactic; if they are used PRN by the patient (even for consecutive days, weeks, or months), they are considered to be acute medications. Patients with previous onabotulinumtoxinA exposure or those with anticipated need for botulinum toxin treatment for any reason during the study (other than study treatment) will be excluded. To continue in the COMPEL study after the 4-week baseline period, participants must have documented ≥15 days of headache per month with headache lasting 4 hours a day or longer. Table 3 summarizes the inclusion and exclusion criteria for COMPEL.

**Table 3:** Summary of main inclusion and exclusion criteria for COMPEL

Inclusion Criteria
Male or female, ≥18 years of age on the day informed consent is signed.
Diagnosis of chronic migraine (≥15 days per month with headache lasting 4 hours a day or longer) with or without medication overuse.
History of chronic migraine (≥3 months).
Patients not taking oral headache prophylaxis medication must have discontinued their oral prophylaxis ≥4 weeks prior to Visit 1.
Patients who are taking oral headache prophylaxis medication must be on a stable dose and regimen of a single oral prophylaxis treatment (prescribed for the use of headache prophylaxis) for at least 4 weeks prior to Visit 2.
If a patient is receiving a headache prophylaxis medication that is specifically prescribed for a non‐headache condition, he or she is eligible to enter the study even if they are receiving another agent on the headache prophylactic list that has been prescribed for headache, as long as the non‐headache medication regimen is stable for at least 4 weeks prior to Visit 1.
Stable medical condition, in the investigator’s opinion.
Ability to follow study instructions (including compliance with a diary) and likely to complete all required visits.
Negative urine pregnancy test at screening and on Day 1 prior to administration of onabotulinumtoxinA (for females of childbearing potential, including premenstrual women).
Documentation of ≥15 days of headache per month with headache lasting 4 hours a day or longer by IVRS patient diary. If fewer than 28 (but more than 20) days are completed in the IVRS patient diary at Day 1, a minimum of 53.6 % of recorded diary days must have a headache lasting 4 hours a day or longer.
Exclusion Criteria
Clinically significant medical condition other than the condition under evaluation (including alcohol/illicit substance abuse, significant pain condition [fibromyalgia, diabetic peripheral neuropathy, etc.]).
Any medical condition that may put the patient at increased risk with exposure to botulinum toxin therapy of any serotype for consistency and safety, including diagnosed myasthenia gravis, Eaton-Lambert syndrome, amyotrophic lateral sclerosis, or any other significant disease that might interfere with neuromuscular function.
Headache diagnosis of chronic tension-type headache (ICHD-III 2.3), cluster headache (ICHD-III 3.1), hypnic headache (ICHD-III 4.9), or hemicrania continua (ICHD-III 3.4).
Headache attributed to another disorder (ie, cervical dystonia, craniotomy, head/neck trauma).
Severe major depressive disorder defined as BDI-II score >24.
Endorsement of a non-zero response on Item 9 (“Thoughts that you would be better off dead, or hurting yourself in some way”) of the PHQ-9 (ie, Item 9 score ≠ 0).
Females who are pregnant, nursing, or are planning a pregnancy during the study.
Females of childbearing potential (including premenstrual women) who are not using a reliable means of contraception.
Previous treatment with botulinum toxin of any serotype for any reason, or immunization to any botulinum toxin serotype.
Anticipated need for botulinum toxin treatment for any reason during the study (other than study treatment).
Known allergy or sensitivity to the study medication or its components.
Previous participation in any botulinum toxin clinical trial.
Concurrent enrollment in an investigational drug or device study or participation in such a study in the 4 weeks immediately prior to Visit 1.
Patient has a condition or is in a situation that, in the investigator's opinion, may put the patient at significant risk, may confound the study results, or may interfere significantly with the patient's participation in the study.
Treatment of study target muscles using acupuncture, TENS, cranial traction, dental splints for headache, nociceptive trigeminal inhibition, occipital nerve block treatments, or injection of anesthetics/steroids within 3 months prior to screening.

BDI-II = Beck Depression Inventory-II; COMPEL = *C*hronic migraine *O*nabotulinu*M*toxinA *P*rolonged *E*fficacy open *L*abel study; ICHD-III = International Classification of Headache Disorders, 3rd edition; IVRS = interactive voice response system; PHQ-9 = Patient Health Questionnaire; TENS = transcutaneous electrical nerve stimulation

### Study treatment and efficacy measures

Patients who meet study criteria will receive 9 onabotulinumtoxinA treatments, administered during Visit 2 (Day 1) through Visit 10 (Week 96). Each treatment will consist of a dose of 155 U of onabotulinumtoxinA administered as 31 fixed-site, fixed-dose intramuscular (IM) injections across 7 specific head and neck muscle areas (Table 4).

**Table 4:** Fixed-dose injection sites for onabotulinumtoxinA

	LEFT Number of units per muscle (number of injection sites^a^)	RIGHT Number of units per muscle (number of injection sites^a^)	TOTAL Number of units per muscle (number of injection sites^a^)
Procerus	—	—	5 (1 site)
Corrugator	5 (1 site)	5 (1 site)	10 (2 sites)
Frontalis	10 (2 sites)	10 (2 sites)	20 (4 sites)
Temporalis	20 (4 sites)	20 (4 sites)	40 (8 sites)
Occipitalis	15 (3 sites)	15 (3 sites)	30 (6 sites)
Cervical Paraspinal Muscle Group	10 (2 sites)	10 (2 sites)	20 (4 sites)
Trapezius	15 (3 sites)	15 (3 sites)	30 (6 sites)
Total Dose	—	—	155 U (31 sites)

Source: [32]

^a^1 injection site = 0.1 milliliter (mL) = 5 U of onabotulinumtoxinA

The International Headache Society Clinical Trials Guidelines Subcommittee for controlled trials of prophylactic treatment of CM in adults [14] recommends that the primary efficacy measure be based on the number of headache days, reported by patient diaries. The primary outcome in COMPEL is the mean change from baseline in the frequency of headache days for the 28-day period ending at Week 108 (following 9 treatments). Secondary efficacy measures will include both mean change from baseline in the frequency of headache days for the 28-day period ending at Week 60 (following 5 treatments) and mean change from baseline in HIT-6 total score over 4 weeks at Week 108 (following 9 treatments) and at Week 60 (following 5 treatments). The remaining measures described are exploratory efficacy measures. Efficacy measures based on daily diary entries and/or baseline measures will include headache frequency, headache duration (ie, whether headache lasts more than 4 hours), headache severity, and abortive headache medications used. Other measures, based on questionnaires completed at Visit 1 (Screening) through Visit 11 (Week 108), will be as follows: HIT-6, MSQ v2.1, Migraine Disability Assessment Questionnaire (MIDAS), Patient Global Assessment of Treatment (PGAT), GAD-7, PHQ-9, PSQI, FSS, 36-Item Short Form Survey Instrument (SF-36), and healthcare resource utilization (ie, headache-related visits to a healthcare professional, headache-related visits to emergency room or urgent care, headache-related admissions/overnights to a hospital, and headache-related diagnostics in past 6 months) [15–21]. Table 5 includes a short description of each measure that will be collected and assessed in COMPEL in addition to headache days.

**Table 5:** Secondary and additional efficacy measures to be collected in COMPEL

Measure	Description
Headache Impact Test (HIT)-6 [33]	• Measures impact of headache and treatment on individual's functional health and well-being.
	• 6 domains: pain; role functioning (ability to carry out usual activities); social functioning; energy or fatigue; cognition; and emotional distress.
Patient Global Assessment of Treatment questionnaire (PGAT)	• Measures impact of treatment on headache symptoms and activities of daily living, including items regarding treatment satisfaction.
Migraine-Specific Quality-of-Life Questionnaire (MSQ) [21]	• Measures health-related quality-of-life impairments attributed to migraine.
	• Divided into 3 domains: role function - restrictive (how migraines limit one’s daily social- and work-related activities), role function - preventive (how migraines prevent these activities), and emotional function (assesses the emotions associated with migraines).
Migraine Disability Assessment (MIDAS) questionnaire [34]	• Assesses number of days that migraine prevented/limited activities in past 3 months, including paid work, education, household work, and non-work activities (family, social, and leisure).
Generalized Anxiety Disorder Assessment (GAD-7) [15]	• Measures frequency with which patients have been bothered by 7 symptoms of anxiety over the previous 2 weeks.
Patient Health Questionnaire 9-item depression scale (PHQ-9) [35]	• Measures frequency with which patients have been bothered by 9 symptoms of depressive disorders over the previous 2 weeks.
Pittsburgh Sleep Quality Index (PSQI) [18]	• Measures the quality and patterns of sleep in 7 areas: subjective sleep quality; sleep latency; sleep duration; habitual sleep efficiency; sleep disturbances; use of sleeping medication; and daytime dysfunction over the previous month.
Fatigue Severity Scale (FSS) [19]	• 9-item measure assessing the severity of fatigue and its impact on daily living.
Short Form Health Survey with 36 questions (SF-36) [20]	• Generic, multipurpose 8-scale profile of functional health and well-being scores as well as psychometrically based physical and mental health measures and preference-based health utility index.
Healthcare Resource Utilization (HRU)	• HRU questionnaire data collected include medication use, healthcare visits, hospitalizations, and emergency room visits.
Assessment of Chronic Migraine Impacts (ACM-I)	• 24 questions about effect of migraines on one’s life, including daily activities, feelings, energy levels, household, leisure activities, social activities, and work, over the previous 7 days.
Assessment of Chronic Migraine Symptoms (ACM-S)	• 12 questions about migraine symptoms over the previous 24 hours, as a series of dichotomous yes/no questions and others that are rated on a numeric rating scale of 0 to 10.

COMPEL = *C*hronic migraine *O*nabotulinu*M*toxinA *P*rolonged *E*fficacy open *L*abel study

### Safety and tolerability measures

A key secondary objective of COMPEL is to evaluate the long-term safety and tolerability (9 treatment cycles) of onabotulinumtoxinA for CM in adult patients. Safety and tolerability will be assessed by the frequency and nature of adverse events (AEs) and physical examinations including vital signs. Spontaneous AEs will be analyzed starting on Day 1 (Visit 2), after injection; analysis will continue through the end of the study period in all patients who receive at least 1 injection of onabotulinumtoxinA. Any patient who exhibits change from baseline in the response to Item 9 of the PHQ-9 (“Thoughts that you would be better off dead, or hurting yourself in some way”) any time during the study will consult with the principal investigator about the suicidal ideation and be withdrawn from the study. Patients who are withdrawn from the study will not receive further protocol-related injections and will be referred to appropriate medical care and follow-up.

If a patient becomes pregnant at any time during the study, she will be withdrawn. Specifically, if a female of childbearing potential becomes pregnant during the study, the patient will not receive any further injections and will be followed for 12 weeks after the last study treatment and at least 4 weeks since the pregnancy was reported, before being exited from the study. The investigator will (1) notify the patient’s physician that the patient was being treated with an investigational drug onabotulinumtoxinA, and (2) follow the progress of the pregnancy until delivery and document the outcome of the pregnancy. Patients who are withdrawn from the study will not receive further protocol-related injections and will be referred to appropriate medical care and follow-up.

### Statistical considerations: sample size and populations

Up to 40 investigative sites in the US, Australia, and South Korea will enroll a sample size (N = 500 patients) that will provide 80 % power to detect subgroup differences of 2.5 or more headache days as the primary endpoint with a 95 % significance level. The safety population (SP) will include all patients who receive at least 1 onabotulinumtoxinA treatment injection.

### Ethical considerations

This study will be conducted in accordance with Institutional Review Board (IRB) regulations (US 21 Code of Federal Regulations [CFR] Part 56.103) or applicable Independent Ethics Committee (IEC) regulations. This protocol is to be conducted in accordance with the applicable Good Clinical Practice (GCP) regulations and guidelines. Written and informed consent will be obtained from each patient or patient’s legally authorized representative prior to enrollment into the study. The Central IRB for the COMPEL Study was reviewed and approved by the Quorum Review IRB in Seattle, WA.

## Discussion

As evidenced by several population-based studies, CM is a debilitating disease with extensive individual and socioeconomic burden [3–5], yet only 33 % of CM patients report taking prophylactic medications [3]. Aside from onabotulinumtoxinA, there are no currently approved prophylactic therapy options specifically for CM. There are four botulinum toxin products approved in the US for other indications; three are serotype A (onabotulinumtoxinA [BOTOX®, Allergan, Inc., Irvine, CA, USA], abobotulinumtoxinA [Dysport®, Ipsen, Paris, France], and incobotulinumtoxinA [Xeomin®, Merz Pharmaceuticals GmbH, Frankfurt, Germany]); and one is serotype B (rimabotulinumtoxinB [Myobloc®/Neurobloc®, Solstice Neuroscience, San Francisco, CA, USA]). Each differs in molecular structure, formulation, and clinical profile. There is no international potency reference standard for botulinum toxins, and each formulation of botulinum toxin is different. Therefore, the units of activity are specific to each product and not interchangeable with those of any other botulinum toxins [22].

Several pharmacological agents have been evaluated in small (≤200 patients) double-blind, placebo-controlled studies for prophylaxis of CM [23–27]. In addition to studying a small population, these were generally short-term trials, with the longest study lasting approximately 7 months [28]. Table 6 presents an overview of the clinical trials that have been conducted in a CM population.

**Table 6:** Clinical trials of prophylaxis pharmacotherapy in chronic migraine

Treatment	Evidence for Use in Chronic Migraine	Number of Patients and Length of Trials
Anticonvulsants:		
Valproate [25, 36]	Small, double-blind, placebo-controlled trials in chronic migraine	N = 70; 12 weeks (Yurekli et al. 2008)
		N = 49; 12 weeks (Bartolini et al. 2005)
Topiramate [27, 28, 37]	Double-blind, placebo-controlled trials in chronic migraine	N = 28; 9 weeks (Silvestrini et al. 2003)
		N = 59; 16 weeks (Diener et al. 2007)
		N = 306; 16 weeks (Silberstein et al. 2007)
Gabapentin [26]	One double-blind, placebo-controlled trial in chronic daily headache	N = 133; 9 weeks (Spira et al. 2003)
Antidepressants:		
Amitriptyline [38]	Small, open-label trial in transformed migraine	N = 27; 9 weeks (Krymchantowski et al. 2002)
Fluoxetine [24]	Small, double-blind, placebo-controlled trial in chronic daily headache	N = 64; 12 weeks (Saper et al. 1994)
Tizanidine [23]	Small, double-blind, placebo-controlled trial in chronic daily headache	N = 134; 12 weeks (Saper et al. 2002)
Botulinum Toxin:		
OnabotulinumtoxinA [29, 30]	Large, double-blind, placebo-controlled trials in CM	N = 679; 56 weeks (Aurora et al. 2010)
		N = 705; 56 weeks (Diener et al. 2010)
Comparison of 2 Therapies:		
OnabotulinumtoxinA vs Topiramate [39, 40]	Small, double-blind, pilot trials in CM	N = 60; 12 weeks (Mathew et al. 2009)
		N = 59; 26 weeks (Cady et al. 2011)

CM = chronic migraine

The PREEMPT clinical program utilized a set of clearly defined diagnostic criteria and outcome measures to assess the safety and efficacy of onabotulinumtoxinA in the prophylaxis of headache in CM patients. PREEMPT consists of the 2 longest CM trials to date (a 24-week, double-blind period followed by a 32-week, open-label phase) [29, 30]. However, this study did not evaluate the effects of onabotulinumtoxinA treatment past 56 weeks [31]. Consequently, the 108-week COMPEL study aims to assess the efficacy, safety, and tolerability of onabotulinumtoxinA as long-term headache prophylaxis in adult patients with CM.

**Fig. 2:**
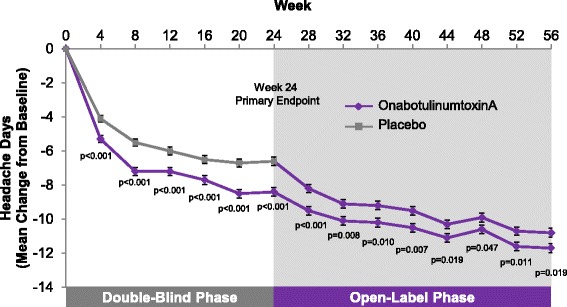
PREEMPT 56-week data showed continued improvement in headache days with onabotulinumtoxinA. Source: [31]. This is a pooled analysis of PREEMPT 1 and 2: the double-blind phase included 688 subjects in the onabotulinumtoxinA group and 696 in the placebo group. Headache days at baseline: 19.9 onabotulinumtoxinA group vs 19.8 placebo group, p = 0.498. PREEMPT = Phase 3 REsearch Evaluating Migraine Prophylaxis Therapy

To our knowledge, COMPEL will be the longest CM trial ever conducted and is expected to add significant knowledge to the literature about long-term use of prophylactic therapy in this patient population. At 15 months, patients who received onabotulinumtoxinA in the PREEMPT study continued to show divergence from those who received placebo during the double-blind phase (Fig. 2); COMPEL will evaluate whether there is continued improvement with onabotulinumtoxinA treatment through 108 weeks, and may or may not demonstrate when a plateau is established. Further, detailed information about the effect of onabotulinumtoxinA on measures other than the primary headache variables will be determined in COMPEL.

Although open-label studies augment knowledge learned from phase 3 randomized controlled trials, they have inherent limitations. One limitation of our study involves its nonrandomized, open-label design. In addition, an open-label design with long-term follow up can be subject to multiple confounders such as unintentional bias, low persistency rates, and concomitant medication changes. Although steps have been taken to control for concomitant medications and headache medication use, stabilization of dose may not always be possible in patients once enrolled. Finally, COMPEL will capture comorbidities in this patient population; however, not all comorbidities will be captured and patients with severe depressive symptoms and suicidal ideation will be excluded.

In conclusion, COMPEL may provide important clinical guidance for physicians by studying treatment outcomes with onabotulinumtoxinA in the presence of oral prophylactics and measuring the effect of onabotulinumtoxinA on a number of exploratory measures, including the assessment of chronic disease comorbidities such as sleep, fatigue, and anxiety. Healthcare resource utilization will be measured, and an extensive number of patient-reported outcomes will be assessed. Through multiple measures, COMPEL may contribute data to improve our understanding of the long-term effects of onabotulinumtoxinA treatment on CM patients.
